# A Quantitative Comparison of Single-Cell Whole Genome Amplification Methods

**DOI:** 10.1371/journal.pone.0105585

**Published:** 2014-08-19

**Authors:** Charles F. A. de Bourcy, Iwijn De Vlaminck, Jad N. Kanbar, Jianbin Wang, Charles Gawad, Stephen R. Quake

**Affiliations:** 1 Department of Applied Physics, Stanford University, Stanford, California, United States of America; 2 Department of Bioengineering, Stanford University, Stanford, California, United States of America; 3 Division of Hematology, Oncology, Stem Cell Transplantation and Cancer Biology, Department of Pediatrics, Stanford University School of Medicine, Stanford, California, United States of America; 4 Howard Hughes Medical Institute, Stanford, California, United States of America; University of Southern California, United States of America

## Abstract

Single-cell sequencing is emerging as an important tool for studies of genomic heterogeneity. Whole genome amplification (WGA) is a key step in single-cell sequencing workflows and a multitude of methods have been introduced. Here, we compare three state-of-the-art methods on both bulk and single-cell samples of *E. coli* DNA: Multiple Displacement Amplification (MDA), Multiple Annealing and Looping Based Amplification Cycles (MALBAC), and the PicoPLEX single-cell WGA kit (NEB-WGA). We considered the effects of reaction gain on coverage uniformity, error rates and the level of background contamination. We compared the suitability of the different WGA methods for the detection of copy-number variations, for the detection of single-nucleotide polymorphisms and for *de-novo* genome assembly. No single method performed best across all criteria and significant differences in characteristics were observed; the choice of which amplifier to use will depend strongly on the details of the type of question being asked in any given experiment.

## Introduction

The recent development of techniques to perform single-cell genome analysis enables direct interrogation of the genetic heterogeneity of cellular populations. Examples of biological phenomena that are accessible for investigation with single-cell sequencing include the clonal diversity within cancer [1]–[3], the role of genetic mosaicism in the biology of multicellular organisms [4]–[6], the genomic variation in gamete cells and embryos [7], [8], and the metabolism of as-yet unculturable microbes [9] [10]–[15].

Whole genome amplification (WGA) is used in order to obtain sufficient material for genetic analyses of DNA isolated from single cells: Illumina and PacBio-based sequencing workflows typically require 1 ng and 500 ng of input material respectively, and a single bacterial or human cell contains on the order of 1 fg or 1 pg of genomic material only. Genome amplification with a factor of 10^3^ to 10^9^ is thus required, depending on the sequencing strategy. WGA can be broadly separated into two categories: temperature-cycled (i.e. PCR-based) methods, and isothermal amplification methods [15]. PCR-based methods rely on ligation of a common primer sequence to sheared DNA, or the use of degenerate oligo-nucleotides for priming. Best-in-class performance for PCR-based methods is achieved with protocols that include a limited MDA pre-amplification phase preceding PCR. The PicoPLEX single-cell WGA kit (NEB-WGA) and the recently described Multiple Annealing and Looping Based Amplification Cycles chemistry (MALBAC) are in this category. The constant region of the primers used in MALBAC is designed such that the products of the initial reaction can form loops, thereby potentially excluding these products as templates for further DNA synthesis [1]. It is unclear to what extent cycling of MDA or loop formation contributes to potential reduction of amplification bias and the performance of MALBAC and NEB-WGA has not been compared systematically.

Isothermal WGA methods, including multiple displacement amplification (MDA) [16], utilize polymerases with high processivity and strand-displacement activity that extend from randomly primed sites. The simplicity of the MDA chemistry makes it relatively straightforward to implement MDA on microfluidic platforms. Improved genomic coverage was reported for MDA in small volumes, but it is unclear what factors contribute to this effect [15], [17], [18].

Although datasets from different WGA methods have been compared [19] in a limited sense, systematic evaluation of the strengths and limitations of each approach on the same samples has been lacking. The present study focuses on single-cell whole genome amplification using MDA, MALBAC and NEB-WGA. We compared numerous metrics of interest, including the specificity (as measured by read mappability), the uniformity of genome coverage, *de-novo* genome assembly quality and the performance of each method for the identification of single-nucleotide variants (SNVs) and the detection of copy number variants (CNVs).

We chose *E. coli* as a target organism because of the relatively low cost of deep sequencing the *E. coli* genome and because amplifications of a bacterial genome from single cells allowed us to study the performance of the different available chemistries in a challenging scenario. Recognizing that the total gain achieved in the amplification reaction is an important parameter, we compared the influence of the gain on the characteristics of interest for the different available methods, where gain is defined as the ratio of DNA output mass over DNA input mass.

## Results

### Design of experiments

**Figure 1:**
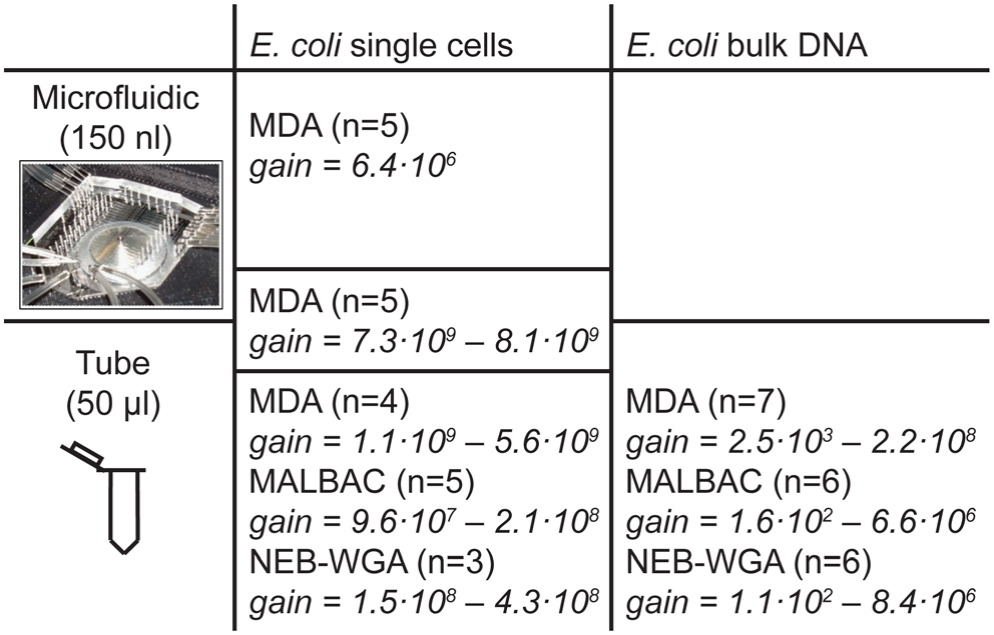
Design of experiments. Overview of experiments, where n denotes the number of experiments of a given type. In the “*E. coli* single cells” column, the box that straddles both the “Microfluidic” and the “Tube” fields corresponds to the method of carrying out a first round of amplification in a microfluidic chamber and then a second round of amplification in a test tube. This method will be denoted by “microfluidic+tube” in subsequent figure captions.

We carried out 41 different reactions based on 8 different experimental designs, distinguished by the DNA template (single *E. coli* cells or *E. coli* bulk DNA), the WGA method (MDA, MALBAC or NEB-WGA), and the volume of the amplification chamber (Fig. 1). To obtain single *E. coli* cells, we sorted individual cells into separate chambers of a microfluidic chip using optical tweezers [20], [21]. Single-cell lysis and WGA reactions were performed on-chip or off-chip as desired. Sequencing of the amplification products was carried out using the Illumina MiSeq 2×250 platform (average depth 158x, minimum 8x, maximum 678x).

### Specificity

**Figure 2:**
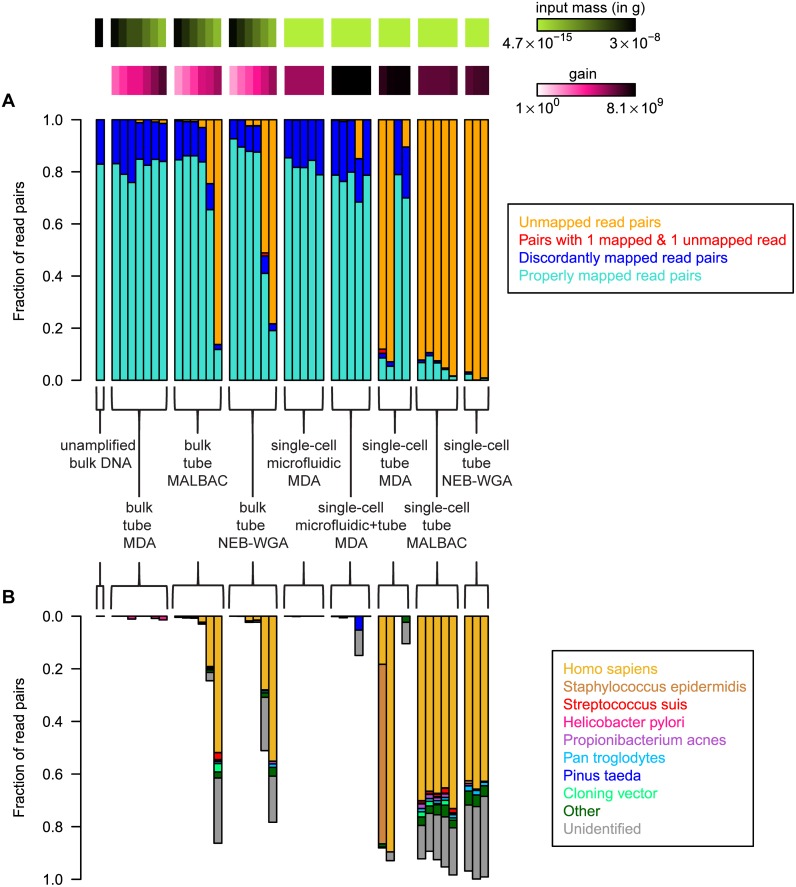
Sequence read classification. (A) Breakdown of read pairs in each experiment according to type of mapping achieved. (B) Breakdown of unmapped reads by organism of origin, expressed as a fraction of the total number of reads.

We investigated specificity by computing the fraction of mapped, unmapped and discordantly mapped read pairs resulting from the different WGA reactions (Fig. 2A). Here, a read pair was flagged as unmapped when both reads in the read pair could not be aligned to the reference genome. A paired-end alignment, i.e. a read pair for which both mates aligned to the reference, was flagged as concordant if the mates were in the expected forward–reverse orientation and had an end-to-end separation ≤2000 base pairs, or as discordant otherwise. The discordant alignments are likely due to chimera formation during the amplification process [17] or during the library preparation.

We found that the fraction of correctly mapped reads is greater (1) for reactions performed in a smaller volume and/or with lower total reaction gain, and (2) for reactions that required fewer hands-on steps (Fig. 2A). We determined the nature of unmapped reads by comparing them against the NCBI database of known nucleotide sequences with the Basic Local Alignment Search Tool, BLAST [22]. On average, 63% of the unmapped reads were assigned to the human genome (36%) or to other known genomes (27%) (see legend Fig. 2B). The remaining unassigned sequences are likely due to the primer-dimer formation during the preparation of sequencing libraries. We found that microfluidic MDA reactions are relatively insensitive to failure due to contamination: the fraction of unmapped reads was very low, even for reactions performed on single *E. coli* cells (mean fraction of unmapped reads 0.035%, n = 5). We attribute this to the small and isolated volume in which the reaction is performed [15]. WGA reactions performed in a tube were more sensitive to background contamination, in particular when the amount of starting genomic material, m_DNA_, was low: MDA, MALBAC and NEB-WGA perform well for m_DNA_≥15 pg, but are unreliable for m_DNA_≤1.5 pg. We found that the PCR-based chemistries, which require many hands-on steps, are particularly sensitive to failure by contamination (fraction of unmapped reads for single-cell NEB-WGA>0.98, n = 3; fraction of unmapped reads for single-cell MALBAC>0.93, n = 5).

### Amplification bias and uniformity

**Figure 3:**
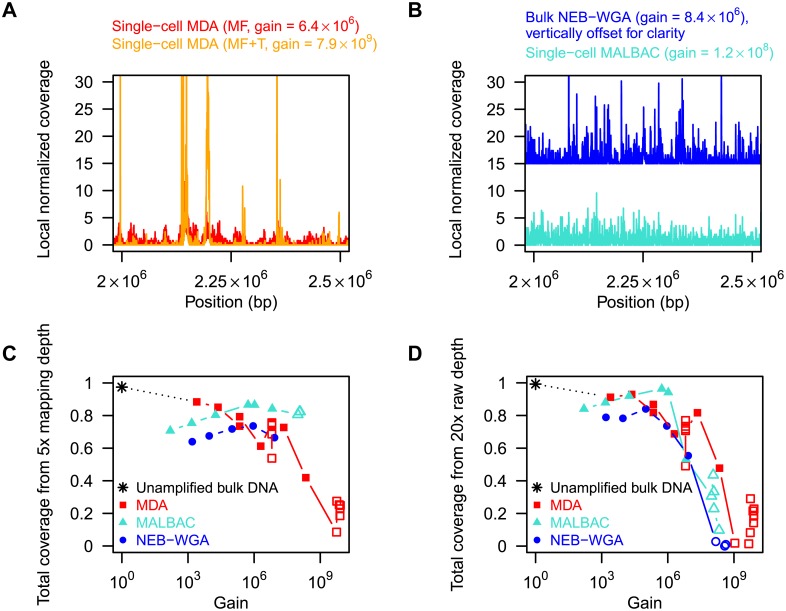
Amplification bias and uniformity. (A) Local mapping density from properly mapped reads (at fixed 5x sampling depth), normalized to average 1, as a function of position along the reference sequence, for single-cell MDA (microfluidic in red, microfluidic+tube in orange). (B) Same as panel A, but for single-cell MALBAC and bulk NEB-WGA. (C) Fractional genome coverage from properly mapped read pairs, plotted as a function of gain. Here, each set of properly mapped read pairs was randomly down-sampled to 5x depth. Experiments that did not generate this many properly mapped reads were not included in the figure. (D) Fraction of the genome covered by mapped read pairs when the set of *raw* read pairs was down-sampled to a fixed depth of 20x, plotted as a function of gain. Filled symbols signify bulk experiments, open symbols single-cell experiments.

We also analyzed the bias in amplification that results from the different chemistries. On average 7% (and up to 45%) of mapped read pairs from a sequencing run were identified as PCR or optical duplicates arising from library construction or repeated reading and were removed using Picard tools [23]. Figures 3A and 3B show examples of the local genome coverage density (normalized to a mean of 1) measured for single-cell MDA, single-cell MALBAC and single-cell NEB-WGA. Consistent with previous results [1], we find that power spectra of the noise in mapping density were distinctly different for MDA and the PCR-based methods, with more low frequency noise contributions for MDA and more high frequency noise contributions for MALBAC and NEB-WGA (see Fig. S1).

To obtain insight into the origin of the amplification bias in MDA, we performed a secondary MDA reaction on the product of a first MDA reaction, and compared the bias before and after the additional round of MDA (Fig. 3A). We found that the bias in amplification that results from the initial reaction is exaggerated by the secondary reaction as regions that were overrepresented in the first reaction generate amplicons at a greater rate in the second reaction. The amplification bias in MDA thus progressively worsens with greater fold amplification [24]. An analysis of the coverage distribution as measured by the Gini index revealed the same dependence of the amplification non-uniformity on gain (see Fig. S2). We next examined the dependence of the genome coverage of reads that mapped to the reference genome as a function of the reaction gain (fixed sampling depth: 5x mapped read pairs; Fig. 3C). We indeed found that the genome coverage is a strong function of the reaction gain in MDA, with greater gain leading to a lower fractional coverage. MALBAC and NEB-WGA however were relatively insensitive to the gain and MALBAC consistently resulted in higher fractional genome coverage than MDA for reactions with a gain greater than 10^6^. Interestingly, we found that isothermal and PCR-based methods performed similarly for reactions with a gain under 10^6^, relevant e.g. for the genome analysis of single human cells (mean fractional coverage 0.82±0.07 for MDA, 0.78±0.07 for MALBAC, 0.69±0.04 for NEB-WGA). We next analyzed the fractional genome coverage achieved given a fixed total sequencing depth (Fig. 3D, 20x sequencing depth), thereby both taking into account reads that mapped and reads that did not map to the reference genome. (The dependence of genome coverage on total sequencing depth is illustrated by the rarefaction curves in Fig. S3.) Remarkably, when considering the fractional coverage at a fixed sequencing depth, we found that the different chemistries perform similarly over the range of reaction gains investigated: the greater inherent uniformity achieved by MALBAC and NEB-WGA (see Fig. 3C) was offset by the larger proportion of unmappable sequences that resulted from these chemistries (Fig. 2A).

### Identification of copy number variants

**Figure 4:**
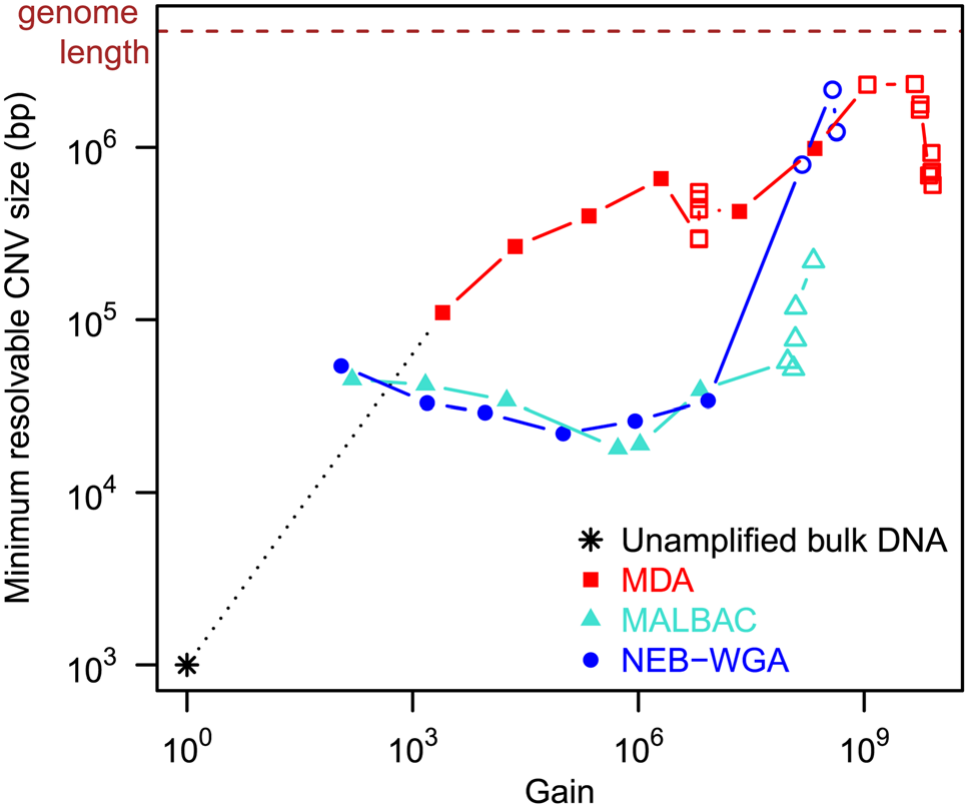
CNV resolution. Size of resolvable duplications (W, minimum width of a sliding window average filter that gives rise to a relative genome mapping density smaller than 2 across all positions in the genome) versus gain. Filled symbols signify bulk experiments, open symbols single-cell experiments.

Biases introduced in whole genome amplification make the robust identification of copy number variants in single-cell sequencing challenging. To obtain insight into the performance of the different chemistries in identifying CNVs, we analyzed the minimal resolvable length of a gene duplication. As a proxy for the minimal resolvable duplication length, we considered the minimum width of a sliding window average filter, W, that gives rise to a relative genome mapping density smaller than 2 across all positions in the genome. Consistent with the above observations, we found that the performance of MDA in the detection of CNVs is strongly dependent on the reaction gain. MALBAC and NEB-WGA are more robust to the effects of gain and outperform MDA for reactions with gain exceeding 2.5•10^3^ (Fig. 4). MALBAC and NEB-WGA performed remarkably similarly in the gain range from 10^2^ to 10^7^, relevant for the analysis of eukaryotic genomes (W = (3.28±1.17)•10^4^ for MALBAC, W = (3.30±1.12)•10^4^ for NEB-WGA). Note that in the case of diploid genomes, using the allele fraction in heterozygous sites may help in detecting CNVs; however, it is unclear whether the effectiveness of such an approach will be compromised in the presence of amplification related allele dropout.

### Error rates

**Figure 5:**
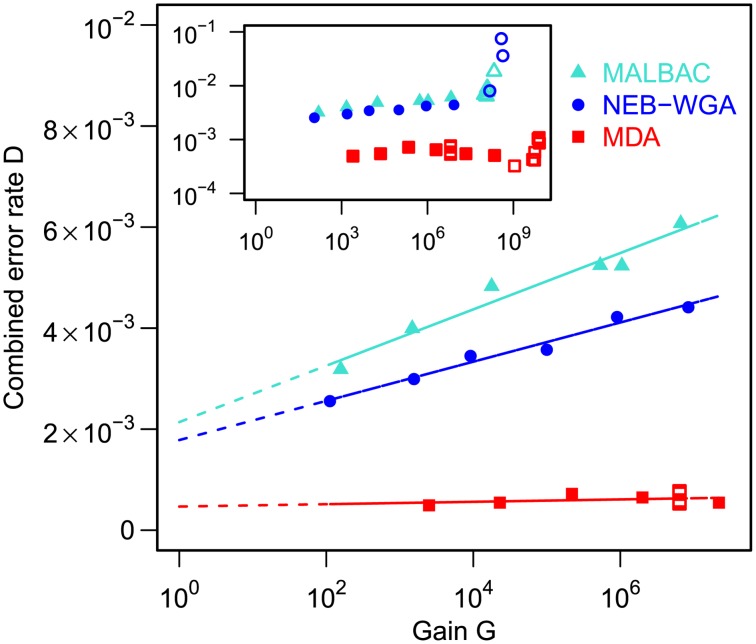
Combined single-nucleotide error rates. Main panel: Experimental error rates D versus gain G, for low gains. Here D is the fraction of bases differing from the reference in the mapped reads. Linear fits for D as a function of 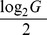 are also shown: their slope approximately indicates the per-base per-cycle replication error rate. Inset: D versus G over the entire gain range. Filled symbols signify bulk experiments, open symbols single-cell experiments.

The rate of single-nucleotide errors introduced in the amplification reaction is another important parameter to consider, in particular for applications where single-nucleotide variants (SNVs) are targeted. For all WGA chemistries considered here, for gains ≤5•10^7^, we found that the combined error rate, D, defined as the fraction of measured bases discrepant from the reference genome, increases logarithmically with the total reaction gain and linearly with the effective number of reaction cycles, computed as 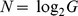. Given a per-base, per-cycle replication error rate, ε, D is expected to scale with N as 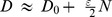 (equation (1), see Appendix S1), where D_0_ is an offset influenced for example by sequencing errors and inaccuracies in the reference. Fitting this model to the data in Fig. 5 for 1≤ gain ≤5•10^7^ allowed us to extract the effective per-cycle error rates for the different WGA chemistries. The offset D_0_ was observed to be roughly of order 10^−3^, consistent with the quality cutoff imposed during quality trimming (Q = 30, corresponding to a per-base error probability of 0.001). We found that the per-base per-cycle error rates for MALBAC and NEB-WGA are similar, 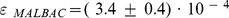 respectively 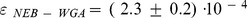.

The per-base error rate for MDA was found to be at least one order of magnitude lower, 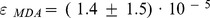. In order to obtain a more precise estimate of the error rate in MDA, we performed error-corrected sequencing using a random barcoding strategy, as described previously [25]. Briefly, DNA molecules were labeled with uniquely identifiable molecular barcodes. Barcoded molecules were subsequently PCR-amplified to generate groups of molecules that carry the same barcode and multiple molecules from each barcode group were sequenced. Consensus calling was then performed to discriminate true variants from sequencing errors. Using this strategy, we achieved a detection limit as low as (9.7±0.4)•10^−7^ per base (determined from unamplified genomic DNA). The measured error rate for MDA at gain ∼3000 was (1.93±0.02)•10^−5^. Estimating the effective number of amplification cycles as log_2_(3000) and using equation (1), we extract the per-cycle per-base error rate to be 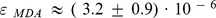, in agreement with reported error rates for the polymerase used in MDA (of order 10^−7^–10^−6^) [16]. The use of a polymerase with proofreading activity and corresponding low error rate is a major advantage of MDA for studies of SNVs.

### Assemblies

**Figure 6:**
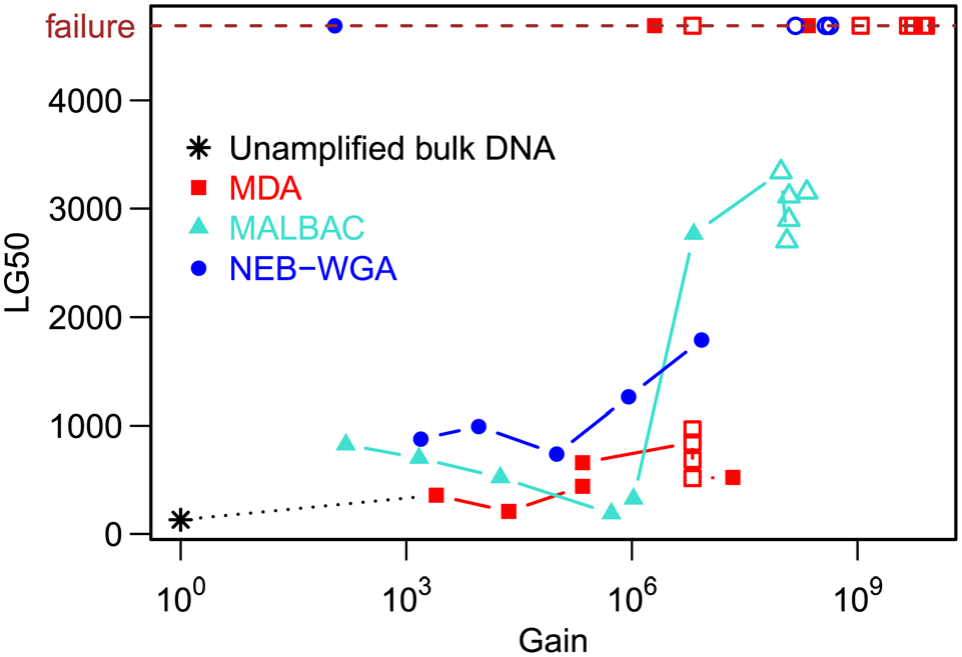
De-novo assemblies. LG50, the minimal number of assembled contigs (≥500 bp) needed to cover 50% of the *E. coli* reference genome, versus reaction gain (at fixed raw sequencing depth 30x). Assemblies that failed to cover 50% of the reference sequence were symbolically assigned the maximum value that LG50 can take in this scenario ((50%•genome length/500) = 4686). Filled symbols signify bulk experiments, open symbols single-cell experiments.

*De-novo* sequence assembly is an important application of single-cell sequencing, for example in studies of novel genomic diversity. We used the SPAdes Genome Assembler 2.5.1 [26] and the quality assessment tool QUAST 2.2 [27] to evaluate the compatibility of the WGA methods with genome assembly. To this end, we investigated LG50, the minimal number of assembled contigs (≥500 bp) required to cover 50% of the *E. coli* reference genome (fixed sequencing depth 30x, Fig. 6). For reactions with gain ≤5•10^6^, isothermal and PCR-based methods performed similarly (mean LG50 416±188 for MDA, 513±262 for MALBAC, 969±223 for NEB-WGA), indicating reconstruction of extended contigs. At higher gains, the assembly quality deteriorated for all three methods. The assemblies resulting from a subset of reactions did not cover 50% of the genome and were marked as “failed” in Fig. 6.

## Discussion

Single-cell sequencing offers a powerful approach for the study of the genomic heterogeneity of cellular populations [2], [28] and for the study of the genetics of microbes that are as-of-yet unculturable [11], [29]. The finite capture efficiency of current sequencing workflows necessitates significant amplification of the target genome. In this work, we have conducted a quantitative performance comparison of the different methods that are available for whole genome amplification. These data measure the differences in performance of temperature-cycled (PCR-based, NEB-WGA, MALBAC) and isothermal amplification (MDA) chemistries, and provide a framework for choosing a WGA chemistry based on the characteristics of interest.

We compared the influence of the gain achieved in WGA on the characteristics of interest. Overall, we found that the performance of WGA chemistries deteriorates with gain, but that not every characteristic and every chemistry is affected to the same extent. The amplification bias in MDA is a direct function of the overall reaction gain, with greater gain leading to greater bias. This observation underlines the importance of tailoring the gain of the amplification reaction to yield the amount of DNA required in a subsequent sequencing workflow. The overall gain can be set through a choice of the volume of the reaction chamber, e.g. by performing the reaction in a small microfluidic reaction chamber, or by limiting the reaction time or the concentration of reagents. It should be noted that we were unable to reproduce the results of a recent report which claimed extraordinarily low bias in single cell amplifications by combining low volume MDA with a subsequent detangling reaction [30]. In contrast to MDA, we found that the inherent bias in amplification in MALBAC and NEB-WGA is not strongly affected by gain. In agreement with previous observations [1], [19], we find that the PCR-based methods lead to a lower long-range variability in read mapping, a property that makes these methods particularly well suited for the detection of CNVs. Within the class of PCR-based amplification chemistries, we found MALBAC and NEB-WGA to compare similarly.

Single-cell WGA reactions are notoriously sensitive to DNA contamination. Contamination is in particular problematic for single-cell studies of small microbial genomes. In general, we found that reactions that entail fewer hands-on steps and that are performed in a smaller reaction volume are more robust against contamination. The relative robustness of amplification reactions in small and enclosed microfluidic environments is an important benefit of microfluidic MDA.

The fractional coverage of the genome, rather than the uniformity and noise spectrum of the mapping density are important for analyses of single-nucleotide variants (SNVs). Our experiments indicated that the inherently low bias in amplification offered by MALBAC and NEB-WGA was offset by the higher sensitivity of these methods to background contamination, leading to a smaller proportion of reads that map to the target genome.

The rate of WGA-induced single-nucleotide errors is another important factor to consider in the context of the detection of SNVs. MALBAC and NEB-WGA displayed an amplification-induced error rate approximately 10 times higher than MDA, which is a consequence of the use of a polymerase with an inherently higher error rate. We conclude that the high coverage at fixed gain as well as the inherently low error rate makes MDA best suited for studies of single-nucleotide variants. While our work here does not investigate the performance of the different amplification chemistries in SNP analyses of diploid genomes, the fundamental error rates we have measured here make strong predictions about the limits of performance for many key metrics in the diploid case.

The assembly of short-read sequence data into a long contiguous sequence is often an important goal in single-cell sequencing projects. We compared the compatibility of different WGA chemistries with genome assembly. We found that MDA, MALBAC and NEB-WGA yielded assemblies of comparable quality (as measured by LG50) for reaction gains ≤5•10^6^. Assembly performance metrics tended to deteriorate at very high gain, consistent with our observation of greater sensitivity to background contamination and lower fractional genome coverage for high-gain reactions.

In conclusion, we have conducted a quantitative assessment of the performance of several widely adopted genome amplification chemistries. The data will enable researchers to design amplification reactions and methods to the needs of their specific experiments.

## Methods

### Bacteria strains and DNA isolation

Invitrogen One Shot TOP10 Chemically Competent *E. coli* were grown overnight in LB broth. These cells were then cleaned in phosphate-buffered saline without CaCl_2_ and MgCl_2_ and either used for single-cell experiments or used for genomic DNA isolation using the QIAamp DNA Mini kit. The gain of the different amplification reactions was modulated by changing the amount of the reaction input material.

### Single-cell primary and secondary MDA

Single *E. coli* cells were sorted in small microfluidic chambers using a laser trap (1 W, 976 nm laser), integrated on a phase-contrast microscope as described previously [20]. Microfluidic devices, attached tygon tubing, and all chemistry not included in the Qiagen REPLIG-g Single Cell MDA kit were U.V. treated for 1 hour. Following U.V. treatment, sorting lines in the device were passivated with phosphate-buffered saline pH 7.4 supplemented with 0.01% pluronic F127 and 0.01% Tween-20 for 15 minutes at room temperature. On the order of 10^4^ *E. coli* cells per µl were loaded into the device and sorted in individual ∼50 nl reaction chambers, partitioned three ways for single-cell MDA chemistry. The first partition, the lysis component, was dead-end filled with 3 nl of a 30 µl volume DLB aliquot supplemented with 3 µl 1 M DTT and 1.4 µl of 10% Tween-20. The entire device was then placed in an incubator (Labnet Mini Incubator, l-5110) at 65°C for 20 minutes, subsequently placed at room temperature for an additional 10 minutes. The second partition, the lysis neutralizing component, was dead-end filled with 3 nl of Stop solution. The last partition, the amplification component, was dead-end filled with ∼43 nl of a 40 µl volume amplification reaction mix aliquot which included 29 µl of single cell REPLI-g reaction buffer, 4 µl of single cell phi29, 2.5 µl of 6.5% Tween-20, and 4.5 µl of 10 mg/ml BSA. The entire device was then placed in the incubator at 31°C for 12 hrs. 20 µl gel pipette tips were then placed in the outlets of the reaction chambers and 5 µl of elution buffer, Tris-EDTA buffer pH 8.0 supplemented with 0.01% Tween-20, was flushed for MDA product retrieval. The single-cell MDA products were then purified with Qiagen MinElute columns and quantified with a Qubit HS DNA kit. Each single-cell reaction generated 40–50 ng of material of which 30 ng was used for Nextera DNA sample library preparation, quantified again with Qubit, and visualized using an Agilent Bioanalyzer 2100. Final libraries were sequenced on the Illumina 2×250 MiSeq platform.

From each single-cell primary MDA reaction, 3 ng was used for a second round of MDA using the same REPLI-g single cell kit. These reactions were performed in a 50 µl volume following the manufacturer’s protocol. Each secondary reaction generated 30–40 µg of total material of which 30 ng was also used for sequence library construction as written above.

### MDA on bulk DNA in tubes

*E. coli* bulk genomic DNA was amplified using the REPLIG-g single-cell kit in 50 µl volume following manufacturer’s protocol (input material 15 ng, 1.5 ng, 0.15 ng, 0.15 ng (bis), 15 pg, 1.5 pg, 0.15 pg). Each reaction generated between 30–40 µg of material. Library construction, material input, and sequencing were carried out as described above.

### NEB-WGA on single-cell and bulk DNA in tubes

Using the integrated optical tweezers, single *E. coli* cells were sorted into separate chambers and retrieved from the microfluidic chip by flushing with elution buffer (described above) in a final volume of 2 µl. The single cells were subsequently amplified with the NEB single cell WGA kit following the manufacturer’s protocol. Single cells on average generated about 1.6 µg of total material. Additionally, six different reactions were performed on bulk genomic *E. coli* DNA (input amounts 15 ng, 1.5 ng, 0.15 ng, 15 pg, 1.5 pg, 0.15 pg). These reactions generated on average 1.5 µg of total material. Final library construction, material input, and sequencing were carried out as described above.

### MALBAC on single-cell and bulk DNA in tubes

Single cells were sorted into single tubes in a 2 µl final volume as described above. Cells were lysed with 1.5 µl Qiagen DLB supplemented with 1 M DTT and heated to 65°C for 10 minutes. Following lysis, 1.5 µl of Qiagen Stop solution was added for final total reaction volume of 5 µl. These lysed cells were then amplified by MALBAC as described previously [1]. First 25 µl of a linear preamplifcation mix was added to 5 µl lysed cell containing 18 µl H_2_O, 3 µl 10x ThermoPol buffer, 1 µl 10 mM dNTP, 1 µl 50 mM MgSO_4_, and 1 µl of 15 mM of each MALBAC Primer (GTGAGTGATGGTTGAGGTAGTGTGGAGNNNNNGGG and GTGAGTGATGGTTGAGGTAGTGTGGAGNNNNNTTT). The reaction was then placed at 94°C for 3 min and immediately quenched on ice. 1 µl of Bst large fragment (NEB 8 U/µl, diluted in 1∶3 in 10X ThermoPol buffer) and 1 µl of PyroPhage3173 DNA Polymerase exo- (Lucigen, 5 U/µl diluted in 4∶25 10X ThermoPol buffer) were added to the reaction and run at 10°C for 45 sec; 20°C for 45 sec; 30°C for 45 sec; 40°C for 45 sec; 50°C for 45 sec; 65°C for 2 min; 94°C for 20 sec, and immediately quenched on ice. Another 1 µl of Bst large fragment (NEB 8 U/µl, diluted in 1∶3 in 10X ThermoPol buffer) and 1 µl of PyroPhage3173 DNA Polymerase exo- (Lucigen, 5 U/µl diluted in 4∶25 10X ThermoPol buffer) were added to the reaction and run at 10°C for 45 sec; 20°C for 45 sec; 30°C for 45 sec; 40°C for 45 sec; 50°C for 45 sec; 65°C for 2 min; 94°C for 20 sec; 58°C for 20 sec and immediately quenched on ice. This last preamplification step was repeated another 5 times. Preamplification reactions were split and PCR amplified with 5 µl 10X ThermoPolbuffer, 1 µl 10 mM dNTP, 3.35 µl 50 mM MgSO_4_, 2 µl Deep Vent Enzyme (NEB, 2 U/µl), 3.33 µl 15 uM MALBAC PCR primer (GTGAGTGATGGTTGAGGTAGTGTGGAG) and supplemented with H_2_O for a final 50 µl reaction volume and run for 17 cycles at 94°C for 20 sec; 59°C for 20 sec; 65°C for 1 min; 72°C for 2 min. Final PCR reactions were then cleaned up using Qiagen MinElute columns and measured using the Qubit HS DNA kit. These single cell reactions generated about 600 ng of material.

Additionally reactions were performed on *E. coli* genomic DNA (input amounts 15 ng, 1.5 ng, 0.15 ng, 15 pg, 1.5 pg, 0.15 pg). The same procedure as detailed above was used for these reactions generating between 900 ng for 0.15 pg starting material to 2 µg for 15 ng starting material. All single-cell and bulk DNA MALBAC reactions were then sheared on a Covaris S2 instrument using the following program: 2 min, 10% duty cycle, intensity 5, 200 cycles/burst and under frequency sweep. Sequencing libraries were then made using the NEBNext DNA Library preparation kit according to the manufacturer’s protocol. Final libraries were quantified with the Qubit HS DNA kit, visualized on an Agilent Bioanalyzer 2100, and sequenced on the 2×250 MiSeq platform.

### Barcoded DNA sequencing

200 ng *E. coli* genomic DNA or bulk MDA products were sheared to 300–500 bp with a Covaris focused-ultrasonicator following manufacturer’s recommendation. Sheared DNA was ligated with pair-end Illumina sequencing adaptors, with 9-bp random barcodes at the beginning of each end. The libraries were PCR amplified to generate redundant molecules and sequenced on Illumina HiSeq 2000 with 2×100 bp mode. Read pairs with the same barcodes were collapsed to generate consensus sequences. At least three reads were required for consensus building. The final duplex consensus sequences were used for error analysis.

### Quality trimming and alignment

MALBAC primers were removed from raw reads with the sequence grooming tool Cutadapt [31] (“cutadapt -n 10 -g GTGAGTGATGGTTGAGGTAGTGTGGAG”, respectively “cutadapt -n 10 -a CTCCACACTACCTCAACCATCACTCAC”). All reads were quality trimmed with Trimmomatic [32], using a 4-base-pair sliding-window algorithm with a quality score cutoff of 30, clipping off ends with at least one occurrence of a quality score below 25, and discarding reads that dropped below a length of 35 base-pairs (“java -jar trimmomatic-0.30.jar PE -phred33 SLIDINGWINDOW:4∶30 LEADING:25 TRAILING:25 MINLEN:35”). Processed reads that were still paired were then aligned to the appropriate *E. coli* strain K12 substrain DH10B reference genome using Bowtie2 [33] in local alignment mode with maximum proper fragment length 2000 (“bowtie2–local –very-sensitive-local -X 2000”). Using Picard tools [23], we removed optical and PCR duplicates (“java -jar MarkDuplicates.jar REMOVE_DUPLICATES = TRUE”) and realigned locally around indels (“java -Xmx4g -jar GenomeAnalysisTK.jar -T IndelRealigner”) over suspicious intervals determined with RealignerTargetCreator (“java -Xmx2g -jar GenomeAnalysisTK.jar -T RealignerTargetCreator”). Processed alignment files were analyzed using SAMtools [34].

### Performance comparison of methods

The nature of unmapped reads was elucidated by comparing them against the NCBI database of known nucleotide sequences with the Basic Local Alignment Search Tool, BLAST [22] (“blastn -db nt -evalue 0.0001 -outfmt ‘6 qseqid sseqid sstart send pident length evalue bitscore sscinames’ -perc_identity 90 -culling_limit 2”). For each read, we quoted the top hit (i.e. the best hit according to e-value) as the organism of origin in Figure 2.

For Figures 3A and 3B, the leftmost mapping positions of reads (considering only the first mate in each pair) were histogrammed in bins of size 250 bp and the resulting mapping density was normalized to a mean of 1 across the genome. For Figures 3C and 3D, the quantity plotted on the vertical axis is the fraction of bases in the genome that were covered at least once by a properly mapped read from the considered number of read pairs.

For Figure 3D, down-sampling to a fixed raw depth 20x was performed by down-sampling the mapped read pairs to F•20x depth (i.e. F•20•(genome length)/(2•250) read pairs), where F≤1 is the fraction of read pairs that were mapped. Similarly, for Figure 6, down-sampling to a fixed raw depth 30x was performed by down-sampling the qualitytrimmed read pairs to T•30x depth, where T is the fraction of read pairs that remained after qualitytrimming. The minimal detectable CNV size W was computed as follows. First, the mapping positions of read pairs were histogrammed in bins of size 250 bp and the mapping density normalized to a mean of 1. Then moving average filters with different window sizes, increasing in steps of 1000 bp, were applied to a circularized (i.e. periodically extended) version of the mapping density. W was the minimum window width such that all windows ≥W gave rise to a relative genome mapping density smaller than 2 across all bins in the genome. The rationale for considering this metric W is that it represents the noise threshold above which a gene duplication could be detected in the smoothed data as a mapping density ≥2.

To compute Gini indices for Figure S2, we first calculated the Lorenz curve (not shown), which is a plot of cumulative share of reads against cumulative share of genome positions covered by those reads, ordered from lowest-covered to highest-covered. [1] The Gini index is then defined as the area between the observed Lorenz curve and the straight Lorenz curve that would result from perfectly uniform coverage. Here, a Gini index of 0 indicates perfect uniformity and a Gini index of 1 indicates maximal non-uniformity.

“Combined single-nucleotide error rates” were calculated by going through each base i in the reference sequence and counting the number of times M_i_ it was matched by a proper alignment and the number of times C_i_ it was contradicted by a proper alignment. The discrepancy ratio is then 
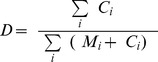
. The base counts necessary for computing M_i_ and C_i_ were extracted from the alignment files using bam-readcount [35], [36]. In computing D, we omitted sites i for which 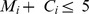 (not enough coverage) and sites i that had 
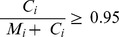
 (typically indicative of a faulty reference base at site i).

*De-novo* assemblies were created using SPAdes 2.5.1 [26] with the *–careful* option and LG50-values were computed using QUAST 2.2 [27]. All sequencing data sets were down-sampled to a fixed (pre-qualitytrimming) depth of 30x using seqtk [37] before assembly. For SPAdes assemblies of bulk DNA, we used k-mer lengths 21, 33, 55, 77, 99, 127; for single-cell assemblies, we used the *–sc* option with k-mer lengths 21, 33, 55. In cases where using SPAdes with the *–sc* option did not give a result but omitting the *–sc* option did, or vice versa, we reported the obtained result regardless of whether the sample was a single cell or bulk DNA. The data points lying on the line labeled “failure” in Figure 6 do not correspond to LG50-values calculated by QUAST, as these assemblies did not cover 50% of the reference genome and so do not have a LG50-value; instead, we artificially assigned the maximum possible LG50-value to these assemblies in order to indicate failure.

Error margins indicated in the present paper correspond to 1 standard deviation.

## Supporting Information

Figure S1 **Power spectra of mapping density.** Mapped reads were down-sampled to 4x depth, and power spectra were smoothed using a moving-average filter with window size 2.13•10^−6^. The MDA, MALBAC and NEB-WGA reactions had gains of the same order of magnitude (2.5•10^3^, 1.5•10^3^ and 1.6•10^3^ respectively). The dashed black lines represent the Lorentzian fits used to extract roll-off frequencies: (4.15±0.06)•10^−5^ bp^−1^ for MDA, (7.94±0.08)•10^−4^ bp^−1^ for MALBAC, (1.32±0.02)•10^−3^ bp^−1^ for NEB-WGA.
(TIF)

Figure S2 **Gini indices of coverage distribution.** Gini index for the distribution of coverage among sites in the genome, plotted as a function of gain. Here, each set of properly mapped read pairs was randomly down-sampled to 5x depth. Experiments that did not generate this many properly mapped reads (e.g. all single-cell NEB-WGA experiments) were not included in the figure. The Gini index is a measure of non-uniformity: a Gini index of 0 indicates perfect uniformity and a Gini index of 1 indicates maximal non-uniformity. By this metric, we found that the mapping uniformity for MDA decreases with reaction gain, whereas the uniformity of PCR-based methods is only a weak function of gain. The amplification bias in PCR-based reactions was lower than the amplification bias in MDA for reactions that required a gain greater than 10^6^.
(TIF)

Figure S3 **Coverage as a function of sequencing depth.** (A) Fraction of genome covered versus sampled depth of mapped read pairs. (B) Same as panel A, but with horizontal axis adjusted by the fraction of raw read pairs that were mapped. We show only the curve that yielded the highest coverage at 20x sequencing depth for each listed experimental category.
(TIF)

Appendix S1 **Modeling discrepancy ratios.**
(DOCX)
