# Supplementary figures and images for: A Quantitative Comparison of Single-Cell Whole Genome Amplification Methods
Source: PLoS One. 2014 Aug 19;9(8):e105585. doi: 10.1371/journal.pone.0105585 (PMC4138190; doi:10.1371/journal.pone.0105585)

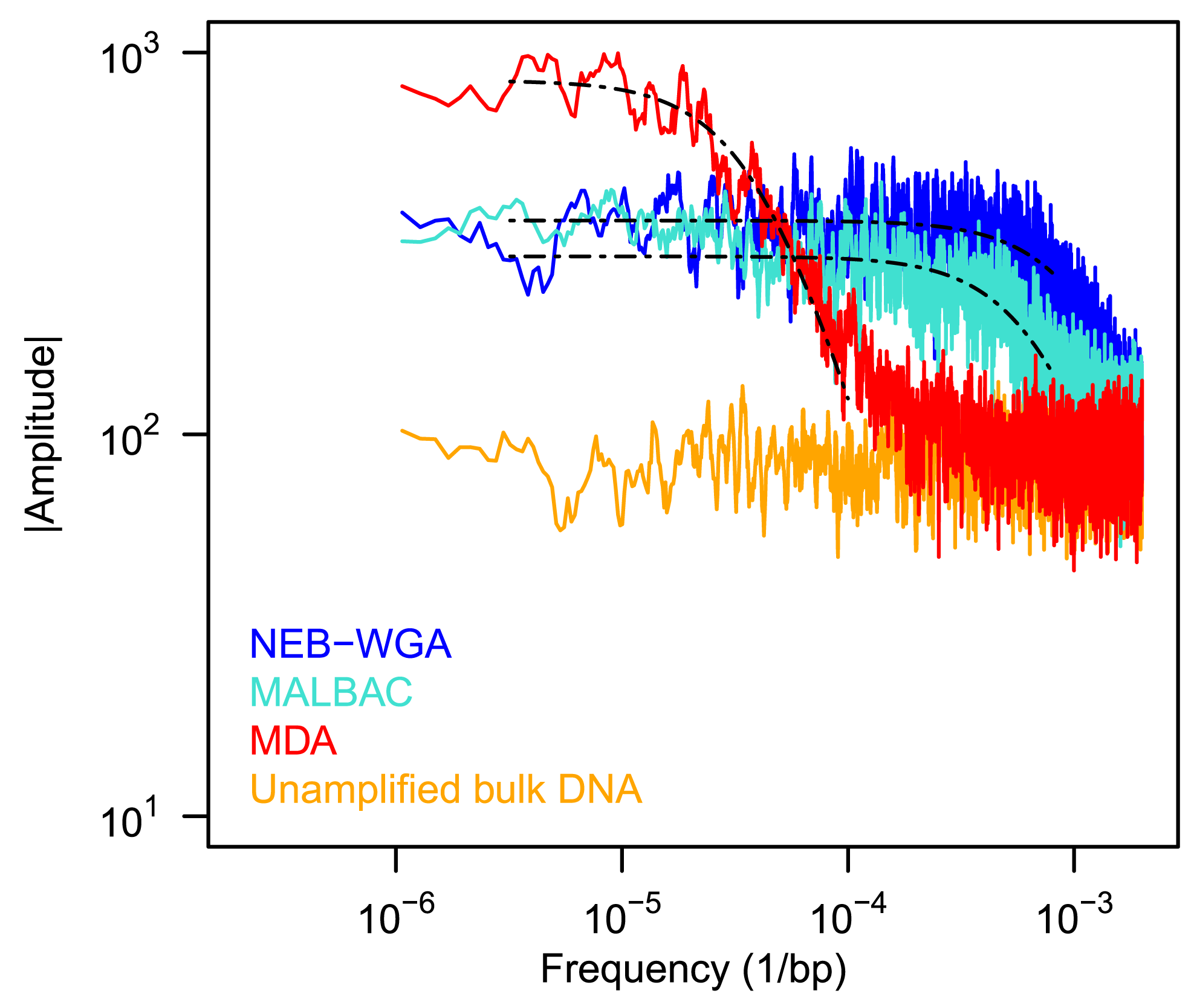

Supplement: Figure S1 — Power spectra of mapping density. Mapped reads were down-sampled to 4x depth, and power spectra were smoothed using a moving-average filter with window size 2.13•10−6. The MDA, MALBAC and NEB-WGA reactions had gains of the same order of magnitude (2.5•103, 1.5•103 and 1.6•103 respectively). The dashed black lines represent the Lorentzian fits used to extract roll-off frequencies: (4.15±0.06)•10−5 bp−1 for MDA, (7.94±0.08)•10−4 bp−1 for MALBAC, (1.32±0.02)•10−3 bp−1 for NEB-WGA. (TIF) [file pone.0105585.s001.tif]

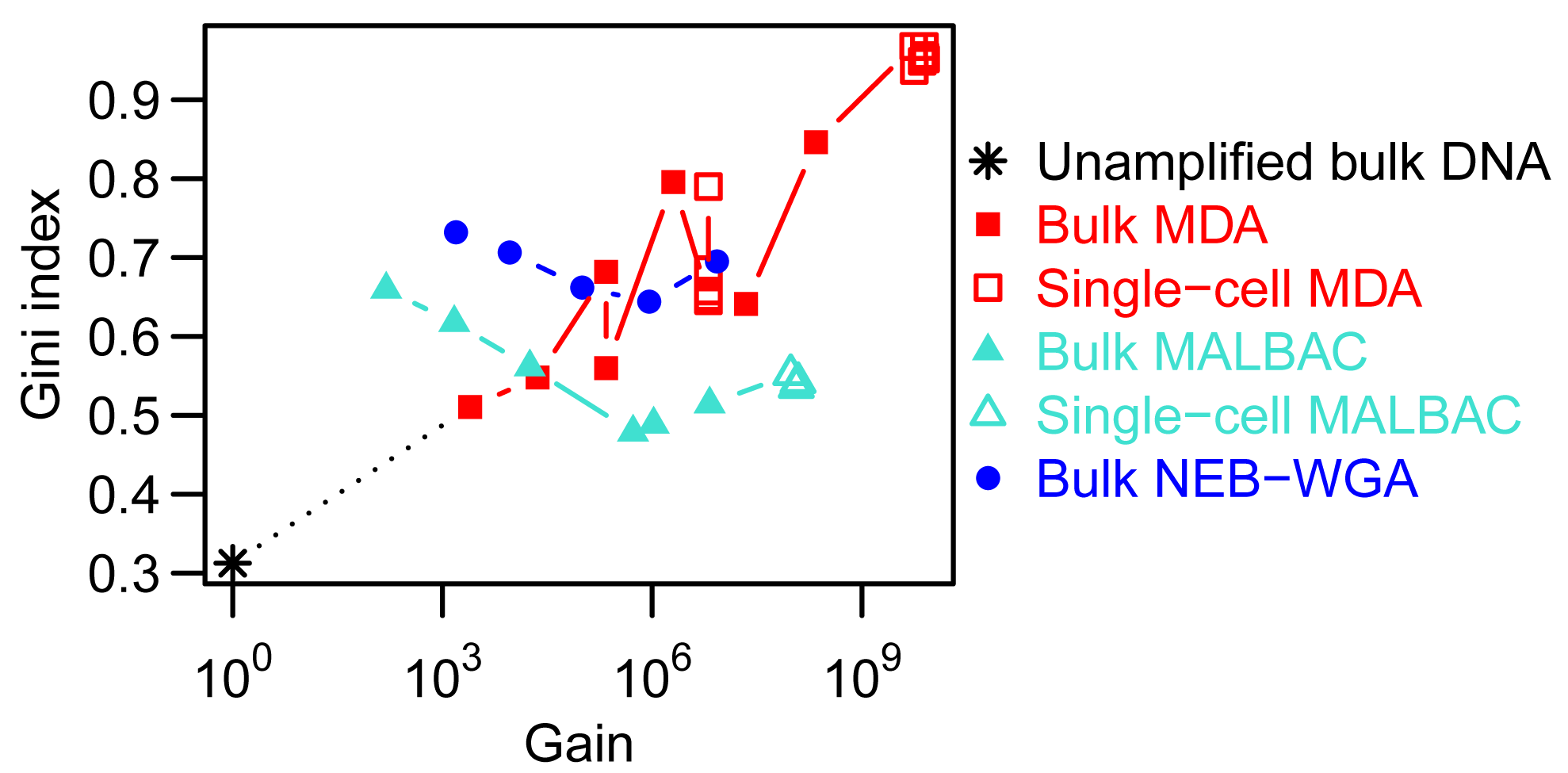

Supplement: Figure S2 — Gini indices of coverage distribution. Gini index for the distribution of coverage among sites in the genome, plotted as a function of gain. Here, each set of properly mapped read pairs was randomly down-sampled to 5x depth. Experiments that did not generate this many properly mapped reads (e.g. all single-cell NEB-WGA experiments) were not included in the figure. The Gini index is a measure of non-uniformity: a Gini index of 0 indicates perfect uniformity and a Gini index of 1 indicates maximal non-uniformity. By this metric, we found that the mapping uniformity for MDA decreases with reaction gain, whereas the uniformity of PCR-based methods is only a weak function of gain. The amplification bias in PCR-based reactions was lower than the amplification bias in MDA for reactions that required a gain greater than 106. (TIF) [file pone.0105585.s002.tif]

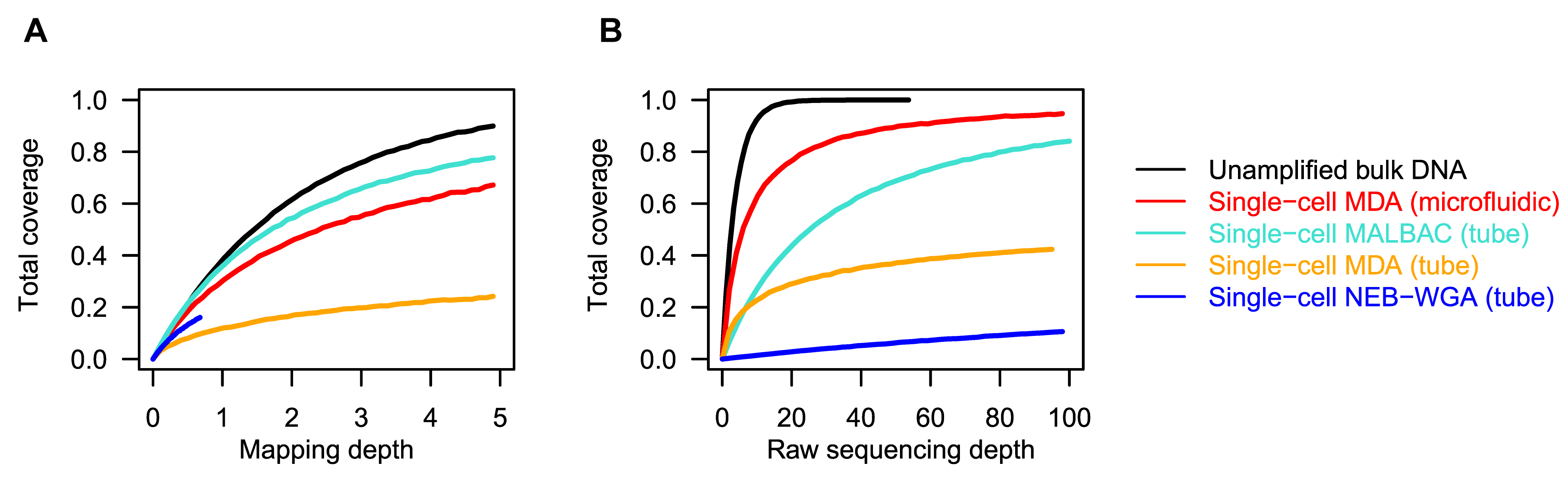

Supplement: Figure S3 — Coverage as a function of sequencing depth. (A) Fraction of genome covered versus sampled depth of mapped read pairs. (B) Same as panel A, but with horizontal axis adjusted by the fraction of raw read pairs that were mapped. We show only the curve that yielded the highest coverage at 20x sequencing depth for each listed experimental category. (TIF) [file pone.0105585.s003.tif]
