# Supplementary material for: A Quantitative Comparison of Single-Cell Whole Genome Amplification Methods
Source: PLoS One. 2014 Aug 19;9(8):e105585. doi: 10.1371/journal.pone.0105585 (PMC4138190; doi:10.1371/journal.pone.0105585)
Supplement: Appendix S1 — Modeling discrepancy ratios. (DOCX) [file pone.0105585.s004.docx]

***Modeling discrepancy ratios (equation (1))***

Let D be the combined error rate (“discrepancy ratio”) observed in an amplification experiment, G the gain, N = log_2_G the number of replication cycles, ε the per-base per-cycle error rate of replication, and K the number of bases in the initial genomic material. After (j-1) cycles, we have 2^j-1^ copies of the original genomic material. In the jth cycle, each one of them is then duplicated, a process in which (2^j-1^∙Kε) new single-base errors are introduced. Here we neglect the probability that the new changes occur in bases that were already altered in a previous cycle; the correction to D obtained when including it would only be of second order in the small quantity ε. Each one of these (2^j-1^∙Kε) errors will be duplicated (N-j) times during the subsequent cycles (cycle number j+1 to cycle number N). Since the total number of bases after N cycles is 2^N^∙K, the final discrepancy ratio is $D=\frac{\sum_{j=1}^{N} 2^{j-1}K\varepsilon\cdot2^{N-j}}{2^{N}K}=\frac{\varepsilon N}{2}$.

Sequencing errors and inaccuracies in the reference genome used for counting discrepancies will add an offset D_0_ to the discrepancy ratio.
